# Oral health of incarcerated young people: a scoping review

**DOI:** 10.1007/s40368-026-01192-0

**Published:** 2026-03-07

**Authors:** M. Mostafa, H. Derbi, S. Kinner, A.M. Laslett, M. Stoové, F. Crombie

**Affiliations:** 1 Melbourne Dental School, University of Melbourne, Melbourne, Australia; 2 Applied Artificial Intelligence Institute, Deakin University, Burwood, Australia; 3 School of Population Health, Curtin University, Perth, Australia; 4 Melbourne School of Population and Global Health, University of Melbourne, Melbourne, Australia; 5 Centre for Alcohol Policy Research, La Trobe University, Melbourne, Australia; 6 Disease Elimination Program, Burnet Institute, Melbourne, Australia; 7 School of Public Health and Preventive Medicine, Monash University , Melbourne , Australia; 8 Australian Research Centre in Sex, Health and Society, La Trobe University , Bundoora, Australia; 9 Justice Health Group, Faculty of Health Sciences, Curtin University, Perth, Australia; 10 Justice Health Group, Murdoch Children’s Research Institute, Melbourne, Australia

**Keywords:** Incarcerated youth, Adolescents, Oral health, Dental caries, Dental health, Correctional facilities health

## Abstract

**Purpose:**

Incarcerated children and adolescents often experience significant disadvantage, heightened health risks, and limited access to healthcare services. Oral health has previously been overlooked in reviews of the health needs of this population. This review identifies and maps evidence on the oral health of incarcerated children and adolescents.

**Methods:**

The review followed PRISMA guidelines for scoping reviews and involved searching five electronic databases (MEDLINE via OVID, CINAHL, Scopus, PsycINFO, and Web of Science) for primary research papers published from January 2000 to April 2025. Search result screening and data extraction were independently undertaken by at least two reviewers.

**Results:**

24 studies, all cross-sectional, were identified for inclusion, with data available from all global regions except Oceania and Africa. Dental caries and periodontal health were the most commonly investigated conditions. Where reported, untreated dental caries prevalence ranged from 42 to 90%; dental trauma prevalence ranged from 11 to 32%. A need for improved oral hygiene and/or professional cleaning was ubiquitous, but periodontitis was rare. Endodontic complications, clinical presence of third molars, and “handicapping” malocclusion were also noted. The developmental defects and wear data were absent.

**Conclusion:**

Research regarding the oral health of incarcerated children and adolescents is sparse and lacks standardised methodologies. The available data indicate a high prevalence of untreated dental disease in these populations and an unmet need for general dental care and potential need for specialist-level dental care in youth detention settings.

**Supplementary Information:**

The online version contains supplementary material available at 10.1007/s40368-026-01192-0.

## Introduction

Children and adolescents detained in correctional facilities are a vulnerable population facing significant health inequalities, including poor oral health outcomes (American Academy of Pediatric Dentistry (AAPD). Management of the Developing Dentition and Occlusion in Pediatric Dentistry 2026). Known behavioural risk factors for oral health problems such as use of tobacco, alcohol, and illicit drugs are prevalent amongst incarcerated young people (Indig et al. 2009; Arora et al. 2019; Borschmann et al. 2020; Casarin et al. 2021). Additional social and ecological risk factors, including experiences of physical and psychological trauma, disrupted family environments, and social disadvantage are also prevalent (Van Harten et al. 2015; Arora et al. 2019). Children and adolescents detained in youth justice facilities often have limited access to both professional dental services and products for dental self-care before and during incarceration (World Health Organization, Regional Office for Europe 2022; Booth et al. 2023; Amaya et al. 2024). Hence, whilst the correctional environment could provide an opportunity for improving or maintaining oral health, it also presents a unique set of challenges.

In alignment with international standards for the treatment of incarcerated young people (United Nations General Assembly 2015), many countries provide some oral health care as part of the services offered. However, standards of healthcare in youth carceral settings vary considerably and are often inadequate (American Academy of Pediatric Dentistry (AAPD). Management of the Developing Dentition and Occlusion in Pediatric Dentistry 2026). Despite oral diseases remaining the most prevalent health conditions across all genders and all World Bank income levels (James et al. 2018; World Health Organization 2022), and studies linking limited access to dental care to a higher burden of oral diseases and poorer oral health-related quality of life (OHRQoL) (Oliveira et al. 2015; Silva et al. 2020), oral health tends to be under-valued by both individuals and systems. Even where there is appropriate investment in correctional healthcare generally, the oral health care provided is often reactive rather than preventive, with treatment reserved for emergencies and late-stage disease interventions such as restorative care or extraction. Consistent oral health screening and oral health promotion are generally lacking compared to other healthcare domains (Bolin and Jones 2006; Alkhadra 2017; Akbar et al. 2022).

Within correctional settings, factors such as extended waiting periods, staff shortages, safety concerns, inadequate facilities, constrained oral health care budgets, and a lack of trust in the healthcare system amongst incarcerated individuals impede the management of conditions such as dental caries and periodontal disease and delivery of comprehensive oral health care services (Ginn 2012; Vandergrift and Christopher 2021; Mussie et al. 2021). The poor oral health of incarcerated youth is largely shaped by socioecological determinants, including low health literacy (amongst youth and caregivers), disrupted family environments, poverty, homelessness, and limited access to dental education and care prior to incarceration (Bolin and Jones 2006; Haysom et al. 2015). These pre-existing vulnerabilities place young people at heightened risk for both incarceration and poor oral health outcomes.

Whilst correctional systems cannot fully address these structural disadvantages, they can play a critical role in mitigating further harm by implementing preventive oral health education and risk reduction strategies upon entry into correctional systems. However, a key challenge remains the lack of continuity in dental care between youth correctional facilities and community services: many justice-involved youth do not receive follow-up oral health care upon release, increasing their risk of worsening long-term oral health outcomes (Bolin and Jones 2006; Haysom et al. 2015; Zorlu et al. 2023). High turnover of youth, with frequent admissions and releases and short incarceration periods, further exacerbates this issue limiting opportunities for timely treatment and preventive interventions.

Despite these challenges, the correctional setting can offer young people access to healthcare that may be less available or inaccessible in the community (Martins et al. 2014; Arora et al. 2019; Amaya et al. 2024). In the absence of existing comprehensive oral health care in many correctional facilities, developing suitable programmes to meet the oral health needs of incarcerated children and adolescents requires first an understanding of what those needs are. Unfortunately, oral health is also an often-overlooked part of health research amongst incarcerated youth; a recent global scoping review of the health of incarcerated adolescents did not capture oral health data (Borschmann et al. 2020). Systematic reviews have reported oral health amongst incarcerated adults to be consistently poor, but the oral health status of incarcerated children and adolescents is less clear (Walsh et al. 2008; Korkosz et al. 2024; Fiegler-Rudol et al. 2025).

This review aims to identify and map available evidence about the oral health status of incarcerated children and adolescents. Given the breadth of the aim in combination with the complexity and heterogeneity of the literature on this topic, a scoping rather than systematic review approach was selected (Munn et al. 2018; Peters et al. 2020).

## Materials and methods

### Reporting format and study registration

The review was reported according to PRISMA guidelines for scoping reviews (Tricco et al. 2018) and is registered on SearchRxiv #20,250,039,453 (10.1079/searchRxiv.2025.00843).

### Eligibility criteria

Studies were eligible if they were published in English in peer-reviewed journals from 1 January 2000 onward. Inclusion criteria were: original primary oral health data reported; data collected from participants during their period detained in a correctional facility; and data presented either limited to, or provided separately for, children and adolescents < 20 years old as defined by the WHO (WHO). Case reports, letters to the editor, non-journal sources, and articles in abstract form only were excluded.

### Search strategy

An electronic search was conducted on five databases: MEDLINE (via OVID), CINAHL, Scopus, PsycINFO, and Web of Science. Search terms related to incarceration and oral health, dental services, tooth disease/s, and dental caries (see Table 1). In some countries, young people are detained in adult facilities; therefore, we did not restrict the search to youth justice settings. The search was initially conducted in August 2024 and finally updated in April 2025. Reference lists of included studies were also screened to identify additional articles.

**Table 1:** Search strategy example in MEDLINE via OVID

Set	Search Statement
1	("Prison*" OR jail* OR detention OR Justice precinct* OR custody OR "correctional facilit*" OR penitentiar* OR incarcerat* OR delinquent* OR inmate* OR convict OR convicts OR offender* OR "juvenile offender*" OR "penal system").mp
2	("Oral health" OR Dental* OR dentist* OR "Dental caries" OR "Tooth disease" OR teeth).mp
3	Mouth diseases/ OR tooth diseases/ OR dental caries/
4	2 OR 3
5	1 AND 4
6	Limit 5 to (English language AND yr = "2000—Current")

### Data collection and analysis

Search results were imported into EndNote X9 (Hupe 2019), and duplicates were removed. Titles and abstracts were independently screened in duplicate by MM and HD or MM and FC, with any uncertainty resolved through discussion. Full-text articles were independently screened by MM, HD and FC, with any conflicts resolved by discussion to achieve consensus. Separate publications that presented different data from the same population sample were included. Data extraction captured authors, date published, number and characteristics of participants, and key findings on oral health. Data were descriptive and classified into three clinical oral health categories (dental caries, periodontal health, and other dental problems) and one combined patient-perspective category (qualitative and quality of life).

Study methodological quality was assessed using the Mixed Method Appraisal Tool (MMAT) (Hong et al. 2018) and classified as low, moderate, or high quality according to the checklist criteria (see supplementary material A). For mixed methods studies, overall quality classification was based on the lowest component evaluation. MMAT evaluation was for the purpose of characterising how research has been conducted and reported; no studies were excluded on this basis. Evaluations were performed in duplicate by MM, HD, AM or FC, with any conflicts resolved by discussion to achieve consensus.

## Results

**Fig. 1:**
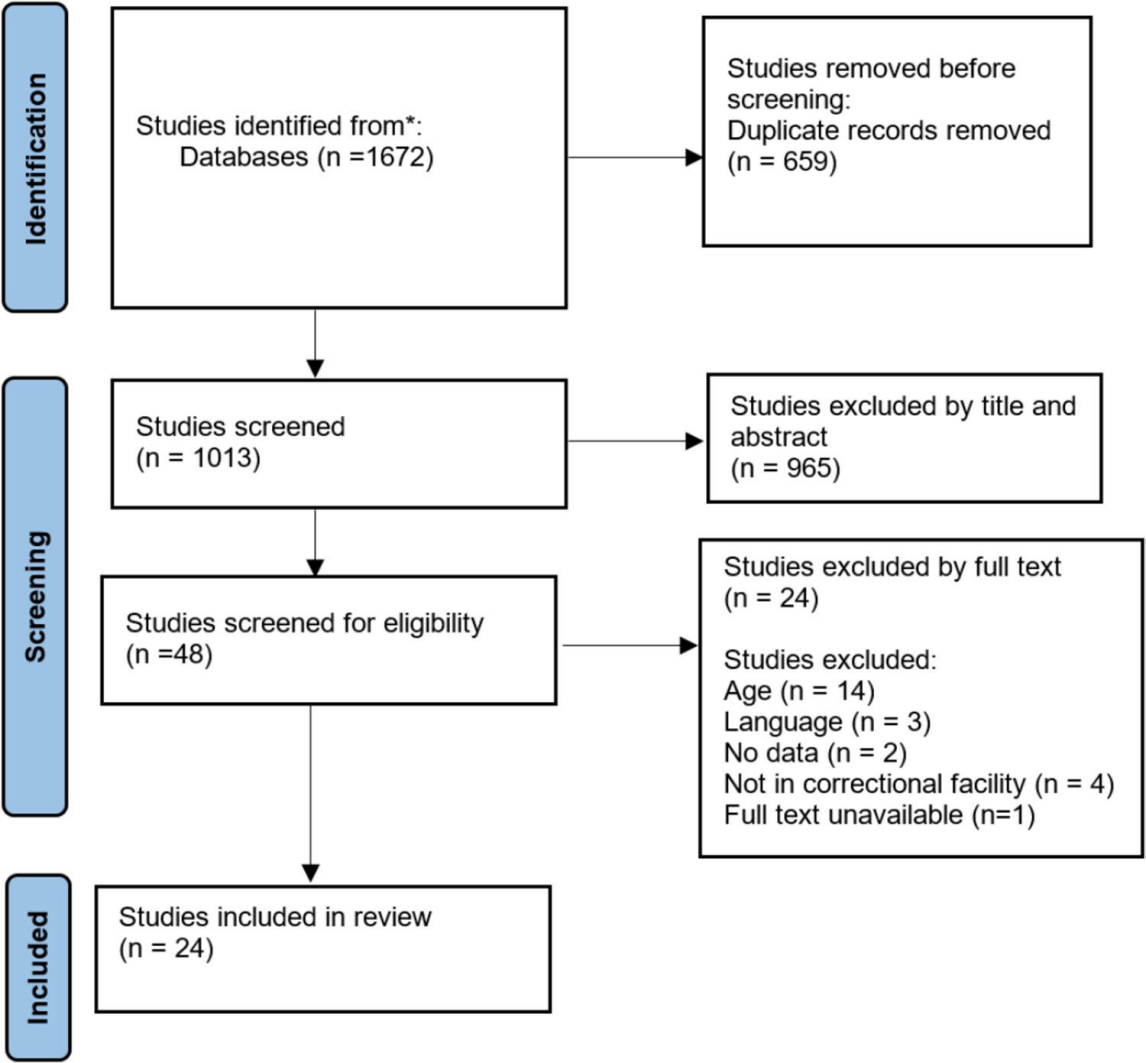
PRISMA flow diagram for the scoping review of oral health requirements of incarcerated children and adolescents

The initial search retrieved 1672 articles. After removing 659 duplicates and 965 articles during title and abstract screening, 48 articles were identified for full-text review. One full-text paper could not be obtained despite attempts to contact the authors directly (Naidoo et al. 2005). Twenty-four articles were finally included in the review (Fig. 1).

### Study characteristics

All included studies were cross-sectional, with six reporting comparative data from a relevant community population (Bolin and Jones 2006; Gaur et al. 2014; Öner Özdaş et al. 2019; Alsaif et al. 2021, 2022; Hwang et al. 2022). Methodological quality was assessed as high for five papers (21%), moderate for 13 papers (54%), and low for six papers (25%); low quality was primarily due to insufficient methodological description (supplementary material B and C).

Sample sizes ranged from 12 to 419, with a total of 2665 participants included across the 24 studies, who ranged from 6 to 19 years old and were overwhelmingly male (Tables 2, 3, 4, 5). Whilst populations from a variety of regions were represented, data came from no more than two countries within each region and one often predominated: South America (8 studies, 7 Brazil), North America (2 studies), Asia (4 studies, 3 India), the Middle East (6 studies, 3 Türkiye and 3 Saudi Arabia), and Europe (4 studies, 3 UK). No studies were from Oceania or Africa.

**Table 2:** Details and results of studies reporting dental caries data

Study Authors Year	Setting Country Detention facility	Population Participant numbers [n]; sex Age	Results				
			Caries prevalence		mean DMFT (SD)	Other data	
			DMFT ≥ 1	DT ≥ 1			
Akbar et al. (2022)	UK (Scotland) Prison	n = 99; 100% male ≤ 20 years old, mean 19.67 SD 0.89	94%		8.10 (4.77)		
Alkhadra (2017)	Saudi Arabia Juvenile detention centre	n = 105; 100% male 12–17 years, mean 16.33	100%		8.92 (4.03)	All DMFT ≥ 6 Mean DMFT increases with age (overlapping CI)	
Alsaif, et al. (2022)	Saudi Arabia Juvenile detention centre	n = 33; 87.9% male 11–14 years, mean 13.5			4.06 (2.397)	Mainstream comparator group (n = 33; mean age 12.8 years) mean DMFT 0.73 (SD 1.04)	
Bolin & Jones (2006)	USA Juvenile detention centre	n = 419; 75.4% male 12–17 years, mean 15.35 SD 1.17	74%	49.6%	3.58 (3.39)	mean DT 1.9 mean MT 0.06 mean FT 1.6 National survey data mean DMFT 2.8	
Casarin et al. (2021) Muniz et al. (2023)Muñoz et al. (2024) Silva et al. (2020)	Brazil Juvenile detention centre	n = 68; 100% male 15–19 years, mean 17.15 SD 1.05	70.6%	42.6%		mean DT 1.06 (SD 1.65), range DT 0–7 mean MT 1.75 (SD 1.98), prevalence 47.1%	
Gaur et al. (2014)	India Juvenile and orphanage home	n = 166; 59% male 6–12 years, mode 11	52.4%			Higher prevalence in boys than girls (54.5% v 50.7%) School comparator group (n = 384; mode 8 years) prevalence 62.1% (64% boys, 59% girls)	
Gökbel and Subaşi Baybuğa (2021)	Türkiye Juvenile penitentiary institution	n = 158; 100% male 13–17 years, mean 16.51 SD 0.86		70.3%		range DT 1–7 prevalence MT 35.4%, range MT 1–6	
Hwang et al. (2022)	South Korea Detention centres	n = 12; 92% male 14–19 years (2% of total study sample)		42%	3.9 (4.5)	mean DT 1.1 (SD 1.8) mean FT 2.8 (SD 3.7) mean MT 0 (SD 0) National survey data mean DMFT 3.7, DT ≥ 1 22% (14–19 years age group 7% of total comparator sample)	
Martins et al. (2014)	Brazil Juvenile detention centre	n = 107; 100% male 13–19 years, mean 16.5 SD 1.3		90%	9.55 (5.17)	mean DT 7.43 (SD 4.80) mean MT 0.49 (SD 1.08) mean FT 1.63 (SD 2.72)	
Morosini et al. (2014) Oliveira et al. (2015)	Brazil Juvenile detention centre	n = 102; 100% male 15–19 years, mean 16.84 SD 0.94	93.1%	77.5%	5.9 (4.5)	range DMFT 0–21 mean DT 2.8 (SD 3.3) mean MT 0.5 (SD 0.9)	
Pallavi et al. (2018)	India Juvenile detention centres	n = 268; 95.1% male 5–20 years			** ≤ 15 years** 0.55 (1.13) **16–20 years** 0.51 (0.97)	** ≤ 15 years (n = 76)** mean DT 0.57 (SD 1.1) mean MT 0.04 (SD 0.19) mean FT 0	**16–20 years (n = 192)** mean DT 0.57 (SD 1.1) mean MT 0.04 (SD 0.19) mean FT 0
Van Harten et al. (2015)	Canada Closed custody youth facility	*n* = 101; 86.1% male 14–20 years, mean and mode 17	81.2%		4.39	mean DT 2.42 mean MT 0.15 mean FT 1.82	
Vinnerljung et al. (2018)	Sweden Secure residential care units	*n* = 85; 50.6% males 13–17 years, median 15.3 male, 16.6 female				“severe” dental decay 43% females, 7% males	
Zorlu et al. (2023)	Türkiye Detention centre	*n* = 232; 100% male 13–19 years, mean 16.65 SD 0.91			6.78 (4.07)	range DMFT 0–23	

DMFT, decayed, missing, and filled teeth; DT, decayed teeth; MT, missing teeth; FT, filled teeth; SD, standard deviation; CI, confidence interval

**Table 3:** Details and results of studies reporting periodontal health data

Study Authors, Year	Setting Country, detention facility	Population Participant numbers [n], sex, age	Results						
			OHI-s						
			Good	Fair	Poor	CPI ≥ 1	Other data		
Argawal et al. (2011)	India; Juvenile detention centre	*n* = 223; 68% male 6–18 years	28%	39%	33%	81%	6–9 years (*n* = 42) CPI score 1 31.0% CPI score 2 45.2% CPI score 3 0%	10–14 years (*n* = 67) CPI score 1 38.8% CPI score 2 43.3% CPI score 3 0%	15–18 years (*n* = 114) CPI score 1 37.7% CPI score 2 32.4% CPI score 3 9.6%
Alkhadra (2017)	Saudi Arabia; Juvenile detention centre	*n* = 105; 100% male 12–17 years, mean 16.33, 74.3% 16–17 years	1%	46%	53%		12–13 years (*n* = 8) mean CI-s 1.7 SD 0.4 mean OHI-s 2.9 SD 0.9	14–15 years (*n* = 19) mean CI-s 2.3 SD 1.2 mean OHI-s 4.4 SD 0.4	16–17 years (*n* = 78) mean CI-s 3.0 SD 0.6 mean OHI-s 5.7 SD 0.2
Bolin & Jones (2006)	USA; Juvenile detention centre	*n* = 419; 75.4% male 12–17 years, mean 15.35 SD 1.17					42% plaque and calculus “to warrant professional clean”		
Gaur et al. (2014)	India; Juvenile and orphanage home	*n* = 166; 59% male 6–12 years, mode 11			Boys 61.36% Girls 58.97%				
Gökbel and Subaşi Baybuğa (2021)	Türkiye; Juvenile penitentiary institution	*n* = 158; 100% male 13–17 years, mean 16.51 SD 0.86					27.2% gingival bleeding		
Oliveira et al. (2015)	Brazil; Juvenile detention centre	*n* = 102; 100% male 15–19 years, mean 16.84 SD 0.94				82.3%	OHI-s ≥ 1 11.5%		
Pallavi et al. (2018)	India; Juvenile detention centres	*n* = 268; 95.1% male 5–20 years	25%	72.4%	2.6%	87.6%	All CPI score max. 1 0.7% CPI score max. 2 85.8% CPI score max. 3 0.4% CPI score max. 4 0.7%	≤ 15 years (*n* = 76) 14.5% OHI-s good CPI score max. 1–2 81.6% CPI score max. 3–4 6.6%	16–20 years (*n* = 192) 29.2% OHI-s good CPI score max. 1–2 67.7% CPI score max. 3–4 19.8%
Van Harten et al. (2015)	Canada; Closed custody youth facility	*n* = 101; 86.1% male 14–20 years, mean and mode 17					5.9% Debris score ≥ 2 2.4% Calculus score ≥ 2		
Vinnerljung et al. (2018)	Sweden; Secure residential care units	*n* = 85; 50.6% males 13–17 years, median 15.3 male, 16.6 female					“mainly pronounced gingivitis” 44% females, 35% males		
Zorlu et al. (2023)	Türkiye; Detention centre	*n* = 232; 100% male 13–19 years, mean 16.65 SD 0.91					mean GI 1.13 (SD 0.47), range GI 0–2.3 mean PI 1.18 (SD0.55), range 0–2.7		

OHI-S, Simplified Oral Hygiene Index; CPI, Community Periodontal Index; CI-s, Simplified Calculus Index; SD, standard deviation

**Table 4:** Details and results of studies reporting other conditions related to oral health

Study Authors, Year	Setting Country, detention facility	Population Participant numbers [n], sex, age	Results							
Malocclusion										
Oliveira et al. (2015)	Brazil; Juvenile detention center	n = 102 100% male 15–19 years (mean 16.8)	DAI > 25 prevalence 60.8%							
Alsaif, et al. (2022)	Saudi Arabia Juvenile detention centre	n = 33; 87.9% male 11–14 years (mean 12.55)	DAI > 25 prevalence 54.5% Mainstream group (n = 33) prevalence 75.8%		30.3% Definite malocclusion/optional treatment 12.1% Severe malocclusion/highly desirable treatment 12.1% Very severe malocclusion/treatment mandatory Mainstream 36.4% Definite, 12.1% Severe, 27.3% Very severe					
Trauma										
Alsaif, et al. (2022)	Saudi Arabia Juvenile detention centre	n = 33; 87.9% male 11–14 years (mean 12.55)	prevalence 27.3% Mainstream group (n = 33) prevalence 6%							
Morosini et al. (2014) Oliveira et al. (2015)	Brazil; Juvenile detention centre	n = 102 100% male 15–19 years (mean 16.8)	prevalence 32.4%							
Öner Özdaş et al. (2019)	Türkiye; Detention centre	n = 231; 100% male mean 16.65 years	prevalence 11.3% Schoolchildren control group (n = 61) prevalence 9.8%							
Pallavi et al. (2018)	India; Juvenile detention centres	n = 268; 95.1% male 5–20 years	mean number of teeth affected				** ≤ 15 years** 0.1 (SD 0.25)		**16–20 years** 0.1 (SD 0.20)	
Pulp complications										
Alsaif, et al. (2022)	Saudi Arabia Juvenile detention centre	n = 33; 87.9% male 11–14 years (mean 12.55)	mean PUFA 0.64 (SD 0.78)							
Pallavi et al. (2018)	India; Juvenile detention centres	n = 268; 95.1% male 5–20 years	5.6% need “pulp care restoration”							
Miscellaneous										
Andrews (2015)	UK (England); Prison & Young Offenders institution	n = 155; 100% male 15–18 years	Third molars partially or fully erupted	**15-year-olds** 38%		**16-year-olds** 22%		**17-year-olds** 48%		**18-year-olds** 27%
Muniz et al. (2023) Muñoz et al. (2024)	Brazil Juvenile detention centre	n = 68; 100% male 15–19 years (mean 17.15)	Halitosis prevalence 51.5% (self-reported) Hyposalivation prevalence 66.2%							
Oliveira et al. (2015)	Brazil; Juvenile detention centre	n = 102 100% male 15–19 years (mean 16.8)	Fluorosis (any severity) prevalence 15.6%							
Pallavi et al. (2018)	India; Juvenile detention centres	n = 268; 95.1% male 5–20 years	Missing (not due to caries) up to 15 years mean 0.01 (SD 0.11) teeth, 16–20 years mean 0.01 (SD 0.10) teeth 2.6% of individuals need extraction (any reason) 32.1% of individuals no treatment need (any reason)							

DAI, Dental Aesthetic Index; PUFA, pulpal involvement, ulceration, fistula, and abscess index; SD, standard deviation

**Table 5:** Details and results of studies reporting Impact of Oral health on quality of life data

Study Authors, Year	Setting Country, detention facility	Population Participant numbers [n], sex, age	Results											
Oral Health Related Quality of Life studies														
			CPQ11-14 mean score		Impact across domains: mean score (SD)									
					Functional limitation			Oral symptoms		Emotional well-being			Social well-being	
Alsaif, et al. (2021) Alsaif, et al. (2022)	Saudi Arabia Juvenile detention centre	n = 33; 87.9% male 11–14 years (mean 12.55)	30.61 (SD 17.70) Mainstream group (n = 33) 9.2 (SD 7.35)		6.45 (5.4) Mainstream 1.30 (2.1)			6.45 (3.5) Mainstream 4.03 (2.7)		9.24 (5.5) Mainstream 0.06 (0.3)			8.45 (7.1) Mainstream 3.82 (5.3)	
			OHIP	Impact across dimensions: % individuals unless otherwise indicated										
			mean score	Functional limitation		Physical pain	Psychological discomfort		Physical disability		Psychological disability	Social disability		Handicap
Barnetche & Cornejo (2017)	Argentina; Juvenile detention centre	n = 70; 94% male 14–18 years	53.37^ (SD 28.77)	79		73	84		74		45	31		26
Martins et al. (2014)	Brazil Juvenile detention centre	n = 107; 100% male 13–19 years (mean 16.5)	reported by subgroup only Min 6.09* (no decay) Max 18.67* (missing teeth)	24.3		67.2	75.7		44.9		50.5	31.7		24.3
Oliveira et al. (2015)	Brazil; Juvenile detention centre	n = 102 100% male 15–19 years (mean 16.8)	6.69* (SD 8.8)	13.7		46.1	42.2		25.5		25.5	14.7		11.8
Silva et al. (2020)	Brazil Juvenile detention centre	n = 68; 100% male 15–19 years (mean 17.15)	13.15* (SD 11.17)	mean score (SD) 1.24 (1.8)		mean score (SD) 2.98 (2.2)	mean score (SD) 3.25 (2.8)		mean score (SD) 1.47 (2.4)		mean score (SD) 1.84 (2.1)	mean score (SD) 1.13 (2.1)		mean score (SD) 1.25 (1.9)
Qualitative studies														
Barnetche & Cornejo (2017)	Argentina; Juvenile detention centre	n = 32; 88% male 14–18 years	Oral condition interferes with obligations as well as social activities Pain amplified by confinement											
Russel et al. (2006)	UK (England); Juvenile detention centre	n = 31; 100% male 15–18 years	Most felt good teeth would have positive effect on self-esteem and job prospects Disagreement regarding impact on attracting partners											

CPQ, Child Perceptions Questionnaire; OHIP, Oral Health Impact Profile (*OHIP-14; ^OHIP-49); min, minimum; max, maximum; SD, standard deviation

Dental caries and periodontal status dominated clinical data, though other oral health conditions such as dental trauma, endodontic complications, malocclusion, hyposalivation, and presence of wisdom teeth were represented (Tables 2, 3, 4). Participants’ self-reported oral health and the impacts of oral health on daily life, well-being, function, and self-esteem were reported in seven papers (Table 5).

### Dental caries

Eighteen studies included dental caries data, though this represented only fourteen distinct population samples as several papers reported on the same population (Table 2). Dental caries was almost universally classified according to the WHO Decayed, Missing, and Filled (DMFT) Teeth Index (Petersen and Baez 2013); however, some papers reported caries prevalence using “rudimentary”, ambiguous or undefined criteria (Gaur et al. 2014; Vinnerljung et al. 2018; Gökbel & Subaşi Baybuğa 2021). Caries experience as measured by DMFT ≥ 1 ranged between 52 and 100%, with mean total DMFT scores varying substantially (Table 2). Where DMFT components were reported separately, these similarly varied substantially between populations, including which component predominated: prevalence of DT ≥ 1 ranged from 42 to 90%. There were no consistent differences in dental caries prevalence by sex or age across studies, and of the four studies including a community population comparison, most reported higher overall caries experience amongst incarcerated children and adolescents with Gaur et al. (2014) being the exception.

### Periodontal Health and Oral Hygiene

Ten studies included periodontal and oral health data. Indices used for periodontal health assessments varied, making the overall determination of prevalence and comparison challenging (Table 3). Most papers used an established index, including the Simplified Oral Hygiene index (OHI-s), the Community Periodontal index (CPI), or a Plaque, Debris, Calculus and Gingival Index (PI, DI, CI and GI) to assess periodontal health; several authors used unspecified criteria for recording the presence of calculus, gingivitis and gingival bleeding (Bolin and Jones 2006; Vinnerljung et al. 2018; Gökbel & Subaşi Baybuğa 2021). High proportions of participants had inadequate oral hygiene, some degree of gingival disease, and calculus formation; however, periodontitis was uncommon, and again, there were no consistent differences in periodontal findings by sex (Table 3). When data reporting and analysis included stratification by age, there were common patterns and significant differences with periodontal health worsening as participants got older, most importantly with regards to prevalence of periodontitis rather than just gingival disease once participants were aged 15 years and above. No studies compared periodontal findings with a non-incarcerated population.

### Other oral health conditions

Eight studies included data on other oral health conditions, though this represented only six distinct population samples as several papers reported on the same population (Table 4). Most studies used an established index or criteria for assessment of dental trauma, malocclusion, and fluorosis; however, except for malocclusion, data lacked severity detail. When compared, there was a higher prevalence of dental trauma in incarcerated populations compared to schoolchildren. Malocclusion was neither more common nor more severe than a community-based cohort. No comparator group data was included for studies reporting on fluorosis, third molar presence, self-reported halitosis or hyposalivation (Andrews 2015; Muniz et al. 2023; Muñoz et al. 2024). Pulp complications were reported for two study populations, again with no comparison to a non-incarcerated group.

### Oral Health-Related Quality of Life and qualitative studies

Six studies presented data on OHRQoL, representing five distinct populations as two papers reported on the same population (Table 5). All studies used validated, age-appropriate instruments: the 14-item Oral Health Impact Profile (OHIP-14) was most common (Martins et al. 2014; Oliveira et al. 2015; Silva et al. 2020), with others selecting the 49-item version (OHIP-49) or the Child Perceptions Questionnaire (CPQ). Studies using OHIP instruments consistently reported greatest impacts in the physical pain and psychological discomfort dimensions (Table 5). The study utilising the CPQ reported greatest impacts in the emotional and social well-being domains and was also the only study to include a comparator group: impact scores were substantially lower amongst this community population overall and for each domain.

Two studies took a qualitative approach conducting either semi-structured individual interviews or focus groups (Russell et al. 2006; Barnetche & Cornejo 2017) (Table 5). Pain was reported as both a major disruptor to normal activities and a driver to access care, including prior to incarceration, but this could also be accompanied by limitations in access or hesitance due to negative perceptions of available dental services. Social impacts varied considerably between individuals, with some unconcerned whilst others reported increased self-consciousness, reduced confidence, and perceived reduced employability.

Girls and young women were particularly under-represented in this data with all but two study populations exclusively male and only four female participants in total.

## Discussion

This scoping review adds to the limited evidence on the oral health status of incarcerated children and adolescents. Consistent with previous reviews of incarcerated populations, high rates of dental caries, poor oral hygiene, and gingival disease were observed (Walsh et al. 2008; Korkosz et al. 2024; Fiegler-Rudol et al. 2025). However, the present review captured additional oral health data, including dental trauma, presence of third molar teeth, and developmental conditions such as malocclusion, which have not been reported in previous reviews of adult incarcerated populations.

Despite this broader scope, the present findings reinforce the considerable evidence gap surrounding the oral health of children and adolescents in custody. This gap remains in part due to the inherent difficulties of conducting independent research in carceral settings: ethical, logistical, and institutional constraints often shape study design, limit sample sizes, and compromise the quality or scope of data collected (Kinner and Young 2018). Included studies were exclusively cross-sectional surveys, serving best to provide preliminary overview data rather than facilitating exploration of causal relationships, associations, or temporal patterns. No radiographic data were available which, although consistent with WHO oral health survey guidelines, will lead to underestimation of the true extent of dental disease (Petersen and Baez 2013). Collapsed reporting, such as encountered for dental trauma and fluorosis, limits existing data utility from both a health status and treatment need perspective (Flores et al. 2007; Andreasen et al. 2012). Common conditions including molar incisor hypomineralisation (MIH) and dental wear were entirely absent from the data. With a prevalence of ~ 13% worldwide (Sluka et al. 2024), MIH would be expected to affect incarcerated child and adolescent populations, as would tooth wear given the possible exacerbation associated with illicit drug use prevalent in incarcerated populations (Da Silva Fonseca et al. 2024).

Current policies within youth justice settings provide limited access to preventive and restorative dental care, often prioritising emergency-based treatment over early intervention and routine care (Martins et al. 2014; NHS 2023). This reactive approach allows minor oral health issues to progress into severe conditions, increasing the need for costly, invasive treatment. Caries prevalence typically peaks in late adolescence/early adulthood (World Health Organization 2022), thus incarceration during this period presents unique and time-limited opportunities to make significant and lifelong improvements in oral health rather than sustaining inequalities for this disadvantaged cohort. Impacts of dental and orthodontic developmental conditions could similarly be mitigated by earlier intervention but may require specialist management (Lygidakis et al. 2022; AAOMS 2024; AAPD 2024, 2025). A data-driven model that integrates routine oral health screenings, targeted preventive care, early restorative treatments, and options for specialist-level care could reduce long-term oral health disparities and support rehabilitation efforts. This would additionally align with broader youth justice reforms that prioritise rehabilitation over punitive approaches by addressing barriers linked to self-esteem, employability, and social reintegration (Agrawal et al. 2011).

There is evidence to suggest that changes in environment and routine during incarceration can positively influence oral health behaviours (Clare 2002; Haysom et al. 2015). Reduced access to sugary snacks and sweetened beverages may be associated with lower rates of new caries lesions in incarcerated populations (Gaur et al. 2014); however, health-related behaviours such as consumption of discretionary foods are often shaped by socioeconomic circumstances, and therefore comparing incarcerated individuals to the general population without accounting for these pre-existing disparities may obscure significant differences in risk profiles. The absence of longitudinal studies further limits any insight into how oral health changes during detention: given incarcerated youth face distinct risks compared to their community-based peers and are significantly more likely to be incarcerated as adults, this would be a valuable knowledge gap for future research to address (Bolin and Jones 2006; Gaur et al. 2014; Öner Özdaş et al. 2019; Alsaif et al. 2021, 2022; Hwang et al. 2022).

The political and systemic prioritisation of security measures over health interventions can act to restrict access to necessary dental care for incarcerated populations. Chronic under-investment in youth correctional healthcare services suggests that oral health remains deprioritised within broader public health and justice systems despite external policy (United Nations 2015; World Health Organization, Regional Office for Europe 2022; Academy and of Pediatric Dentistry (AAPD), 2025; ; ). Addressing availability and accessibility of services is not sufficient: effective care requires utilisation of services and further investigation of barriers from this perspective is also warranted. A reading of the papers included in this scoping review revealed fear of the dentist, negative perceptions of dentists generally and prison dentists particularly, and high rates of never having engaged with dental care. Low prioritisation of dental health has been identified as a major contributing factor to non-attendance for young people in custody (Morosini et al. 2014; Haysom et al. 2015).

Even if the opportunities incarceration presents for improving oral health can be effectively realised, without stronger post-release pathways these benefits may not be sustained. The broader psychosocial implications of poor oral health in incarcerated youth remain understudied but can both be profound and impair reintegration into society post-incarceration (Arora et al. 2020), making a coordinated approach with community dental services essential to support not only better dental outcomes but general rehabilitation (Ginn 2012). Suggestions for coordinated and integrated systems are not new (Conte 1981), and some “successes” have been reported when prison healthcare was transferred to community services (Ginn 2012).

### Limitations

The present review had several limitations. First, studies published before the year 2000 were excluded to provide a contemporary snapshot of the oral health of incarcerated children and adolescents. Although the Global Burden of Disease reports began including oral health data in 1990, this decision was based on the expectation that more recent studies would better capture contemporary health outcomes and is in keeping with other recent reviews in the field (Walsh et al. 2008; World Health Organization 2022; Korkosz et al. 2024). Second, the search was limited to papers published in English and consequently excluded three studies; one each from Spain, Thailand, and South Korea (Oliván Gonzalvo 2002; Rungrasmeetaweemana 2014; Jeon and Baek 2016). Whilst their exclusion may have resulted in the lack of some regional insights, the overall review findings align with the English language abstracts of these papers as well as broader international evidence. Finally, only peer-reviewed published data were included: grey literature and internal institutional documentation may capture additional data or details, but is difficult to systematically identify, non-standardised in reporting format, and access is often restricted.

Generalisability of results is a common concern, and certainly in the present scoping review there was considerable methodological heterogeneity which impeded any comparative or combined analysis. Whilst improved standardisation would facilitate comparison, variability in disease prevalence and patterns is likely unavoidable: reflecting known inequalities in oral health burden across populations especially along social disadvantage lines (World Health Organization 2022), as well as differences in health systems. This emphasises the need for population-specific research and representative sampling prior to decision-making. For example, whilst males dominate both this review and incarcerated populations there is evidence that females in detention may have distinct health risks and needs: these include differences related to reproductive health, diet, and drug use disorder rates (Herbert et al. 2012; Fazel et al. 2017; World Health Organization, Regional Office for Europe 2022). Similarly, the geographic imbalance represented in the review data limits generalisability and highlights the potential for unexamined global disparities.

It is recommended that future research should prioritise standardised, comprehensive, and context-specific data collection. Longitudinal and interventional study designs, including exploration of how oral health care can be effectively delivered during and after incarceration, are suggested to inform evidence-based policies and practices for this vulnerable and underserved population.

## Conclusion

The existing evidence on the oral health of incarcerated children and adolescents reports primarily on dental caries and gingival conditions, identifying a considerable but variable burden of untreated disease including periodontitis in those 15 years of age and older. Other oral health conditions were rarely assessed and generally reported in limited detail. Where available, community group data comparison trended towards a higher prevalence of acquired, but not developmental, dental conditions and greater OHRQoL impacts for incarcerated children.

## Supplementary Information

Below is the link to the electronic supplementary material.

Supplementary file1 (PDF 406 KB)

## Data Availability

No datasets were generated or analysed during the current study.
