# Supplementary material for: Oral health of incarcerated young people: a scoping review
Source: Eur Arch Paediatr Dent. 2026 Mar 7;27(4):1007–20. doi: 10.1007/s40368-026-01192-0 (PMC13461829; doi:10.1007/s40368-026-01192-0)
Supplement: Supplementary file 1 [file 40368_2026_1192_MOESM1_ESM.pdf]

Supplementary material

A) Mixed Methods Appraisal Tool (MMAT) Key

Overall quality rating according to the evaluation by the applicable study design checklist*

High: Applies if all or most criteria from the checklist are met; if any criteria are not met, it is considered very unlikely that the study or review conclusions would change.

Moderate: Applies if some of the checklist criteria are met; if any criteria are not met or are inadequately described, it is considered unlikely to significantly affect the conclusions.

Low: Applies if few or none of the checklist criteria are met; if criteria are not fulfilled or are inadequately described, the conclusions are considered likely or very likely to change.

*****Mixed methods studies were evaluated against each applicable checklist with the overall quality determined by the lowest rated evaluation, based on the premise that a combination cannot exceed the quality of its weakest component.

B) Summary table of included studies, MMAT study category and methodological quality rating.

Study	MMAT Study Category	Rating
Agrawal et al., 2011	Quantitative descriptive	Moderate
Akbar et al., 2022	Quantitative descriptive	Moderate
Alkhadra, 2017	Quantitative descriptive	Moderate
Alsaif, et al., 2021	Quantitative descriptive	Moderate
Alsaif, et al., 2022	Quantitative descriptive	Moderate
Andrews, 2015	Quantitative descriptive	High
Barnetche & Cornejo, 2017	Mixed methods	Low
Bolin & Jones, 2006	Quantitative descriptive	Low
Casarin et al., 2021	Quantitative descriptive	Low
Gaur et al., 2011	Quantitative descriptive	Low
Gökbel Sönmez & Subaşi Baybuğa 2021	Quantitative descriptive	Low
Hwang et al., 2022	Quantitative descriptive	Moderate
Martins et al., 2014	Quantitative descriptive	Moderate
Morosini et al., 2014	Quantitative descriptive	High
Muniz et al., 2022	Quantitative descriptive	High
Muñoz et al., 2024	Quantitative descriptive	High
Oliveria et al., 2015	Quantitative descriptive	Moderate
Öner Özdaş et al., 2018	Quantitative descriptive	Moderate
Pallavi et al., 2018	Quantitative descriptive	Moderate
Russel et al., 2006	Qualitative	Moderate
Silva et al., 2021	Quantitative descriptive	High
Van Harten et al., 2015	Quantitative descriptive	Moderate
Vinnerljung et al., 2018	Quantitative descriptive	Low
Zorlu et al., 2023	Quantitative descriptive	Moderate

C) MMAT methodological quality criteria evaluations per included study

Agrawal et al., 2011

	Methodological quality criteria	Responses			
		Yes	No	Can't tell	Comments
Screening questions	S1. Are there clear research questions?	x			
	S2. Do the collected data allow to address the research questions?		x		Not for "dental diseases"; data collected limited to oral hygiene & periodontal health
Quantitative descriptive	4.1. Is the sampling strategy relevant to address the research question?	x			Convenience volunteer sample
	4.2. Is the sample representative of the target population?			x	Participation rate unknown
	4.3. Are the measurements appropriate?			x	Yes for OHI-S & CPI clinical data; very likely no for oral hygiene practice
	4.4. Is the risk of nonresponse bias low?			x	Details of non-participants not provided beyond "ill and uncooperative excluded"
	4.5. Is the statistical analysis appropriate to answer the research question?	x			When taken as limited to OHI-S and CPI data

Akbar et al., 2022

	Methodological quality criteria	Responses			
		Yes	No	Can't tell	Comments
Screening questions	S1. Are there clear research questions?	x			
	S2. Do the collected data allow to address the research questions?	x			To some extent: caries risk factor assessment not comprehensive and some questionnaire data rudimentary
Quantitative descriptive	4.1. Is the sampling strategy relevant to address the research question?	x			Convenience volunteer sample
	4.2. Is the sample representative of the target population?			x	Participation rate unknown
	4.3. Are the measurements appropriate?	x			Questionnaire and clinical data limitations not-withstanding
	4.4. Is the risk of nonresponse bias low?			x	No details of eligible non-participant attributes
	4.5. Is the statistical analysis appropriate to answer the research question?	x			

Alkhadra, 2017

	Methodological quality criteria	Responses			
		Yes	No	Can’t tell	Comments
Screening questions	S1. Are there clear research questions?	x			
	S2. Do the collected data allow to address the research questions?	x			
Quantitative descriptive	4.1. Is the sampling strategy relevant to address the research question?			x	Sampling strategy unclear
	4.2. Is the sample representative of the target population?			x	Participation rate unknown
	4.3. Are the measurements appropriate?	x			
	4.4. Is the risk of nonresponse bias low?			x	Details of non-participants not provided beyond “ill and uncooperative excluded”
	4.5. Is the statistical analysis appropriate to answer the research question?	x			

Alsaif, et al., 2021

	Methodological quality criteria	Responses			
		Yes	No	Can’t tell	Comments
Screening questions	S1. Are there clear research questions?	x			
	S2. Do the collected data allow to address the research questions?	x			
Quantitative descriptive	4.1. Is the sampling strategy relevant to address the research question?	x			Convenience volunteer sample
	4.2. Is the sample representative of the target population?			x	Participation rate unknown; small numbers
	4.3. Are the measurements appropriate?	x			
	4.4. Is the risk of nonresponse bias low?			x	Details of non-participants not provided beyond “unable to participate and respond to the study due to mental or physical disability” excluded
	4.5. Is the statistical analysis appropriate to answer the research question?	x			

Alsaif, et al., 2022

	Methodological quality criteria	Responses			
		Yes	No	Can't tell	Comments
Screening questions	S1. Are there clear research questions?	x			
	S2. Do the collected data allow to address the research questions?	x			
Quantitative descriptive	4.1. Is the sampling strategy relevant to address the research question?	x			Convenience volunteer sample
	4.2. Is the sample representative of the target population?			x	Participation rate unknown; small numbers
	4.3. Are the measurements appropriate?	x			
	4.4. Is the risk of nonresponse bias low?			x	Details of non-participants not provided beyond "unable to participate and respond to the study due to mental or physical disability" excluded
	4.5. Is the statistical analysis appropriate to answer the research question?	x			

Andrews, 2015

	Methodological quality criteria	Responses			
		Yes	No	Can't tell	Comments
Screening questions	S1. Are there clear research questions?	x			
	S2. Do the collected data allow to address the research questions?	x			
Quantitative descriptive	4.1. Is the sampling strategy relevant to address the research question?	x			Convenience volunteer sample
	4.2. Is the sample representative of the target population?			x	Likely but not explicitly stated; nearly 40% of total institute population
	4.3. Are the measurements appropriate?	x			
	4.4. Is the risk of nonresponse bias low?			x	Unclear acceptance rate/non-participant rate or attributes
	4.5. Is the statistical analysis appropriate to answer the research question?	x			

Barnetche & Cornejo, 2017

	Methodological quality criteria	Responses			
		Yes	No	Can't tell	Comments
Screening questions	S1. Are there clear research questions?	x			
	S2. Do the collected data allow to address the research questions?	x			For quantitative; not obvious for qualitative
Qualitative	1.1. Is the qualitative approach appropriate to answer the research question?	x			
	1.2. Are the qualitative data collection methods adequate to address the research question?	x			Some aspects of method not explicit but indications present
	1.3. Are the findings adequately derived from the data?		x		Some data collected not reported, stated methodology not obviously used for findings
	1.4. Is the interpretation of results sufficiently substantiated by data?		x		Stated methodology not obviously used and inadequately substantiated by quotes
	1.5. Is there coherence between qualitative data sources, collection, analysis and interpretation?		x		Sampling and/or data saturation unclear, actual analysis and findings not obviously matched to stated methodology
Quantitative descriptive	4.1. Is the sampling strategy relevant to address the research question?	x			Convenience volunteer sample
	4.2. Is the sample representative of the target population?			x	Participation rate unknown
	4.3. Are the measurements appropriate?	x			
	4.4. Is the risk of nonresponse bias low?			x	Unclear acceptance rate/non-participant rate or attributes
	4.5. Is the statistical analysis appropriate to answer the research question?	x			

Bolin & Jones, 2006

	Methodological quality criteria	Responses			
		Yes	No	Can't tell	Comments
Screening questions	S1. Are there clear research questions?	x			
	S2. Do the collected data allow to address the research questions?		x		Data too limited to assess "dental disease"
Quantitative descriptive	4.1. Is the sampling strategy relevant to address the research question?	x			Record review with sample size calculation
	4.2. Is the sample representative of the target population?			x	Random selection without comment on this
	4.3. Are the measurements appropriate?		x		Yes for DMFT; other subjective/unclear
	4.4. Is the risk of nonresponse bias low?	x			Random selection
	4.5. Is the statistical analysis appropriate to answer the research question?			x	Yes for DMFT; other data unclear/absent

Casarin et al., 2021

	Methodological quality criteria	Responses			
		Yes	No	Can't tell	Comments
Screening questions	S1. Are there clear research questions?	x			
	S2. Do the collected data allow to address the research questions?		x		Yes for tooth loss; significant omissions for associated factors
Quantitative descriptive	4.1. Is the sampling strategy relevant to address the research question?	x			Convenience volunteer sample
	4.2. Is the sample representative of the target population?	x			Highly likely as 92% participation rate
	4.3. Are the measurements appropriate?		x		Major associated factors for tooth loss not considered
	4.4. Is the risk of nonresponse bias low?	x			92% participation rate
	4.5. Is the statistical analysis appropriate to answer the research question?	x			Within limitations of data collected

Gaur et al., 2011

	Methodological quality criteria	Responses			
		Yes	No	Can't tell	Comments
Screening questions	S1. Are there clear research questions?	x			
	S2. Do the collected data allow to address the research questions?	x			
Quantitative descriptive	4.1. Is the sampling strategy relevant to address the research question?			x	Limited details for community comparator group only
	4.2. Is the sample representative of the target population?			x	
	4.3. Are the measurements appropriate?			x	Inadequate details for habit data
	4.4. Is the risk of nonresponse bias low?			x	No or limited sampling details
	4.5. Is the statistical analysis appropriate to answer the research question?		x		Descriptive with no reporting on claimed correlation results; format of results data differed to research questions (objectives)

Gokbel Sonmez & Subasi Baybuga, 2021

	Methodological quality criteria	Responses			
		Yes	No	Can't tell	Comments
Screening questions	S1. Are there clear research questions?	x			
	S2. Do the collected data allow to address the research questions?		x		Unfocussed and extremely broad research question; critical methodological and data details lacking
Quantitative descriptive	4.1. Is the sampling strategy relevant to address the research question?	x			Convenience volunteer sample
	4.2. Is the sample representative of the target population?			x	~60% response rate
	4.3. Are the measurements appropriate?		x		Rudimentary for dental status and limited direct applicability to care needs beyond some dental acre required; general health seem likely similarly inadequate for purpose of determining "health care needs" in meaningful way
	4.4. Is the risk of nonresponse bias low?			x	No details of eligible non-participant attributes
	4.5. Is the statistical analysis appropriate to answer the research question?		x		

Hwang et al., 2022

	Methodological quality criteria	Responses			
		Yes	No	Can't tell	Comments
Screening questions	S1. Are there clear research questions?	x			
	S2. Do the collected data allow to address the research questions?	x			
Quantitative descriptive	4.1. Is the sampling strategy relevant to address the research question?	x			Record audit
	4.2. Is the sample representative of the target population?			x	Sampling strategy not specified
	4.3. Are the measurements appropriate?	x			
	4.4. Is the risk of nonresponse bias low?			x	Sampling strategy not specified
	4.5. Is the statistical analysis appropriate to answer the research question?	x			

Martins et al., 2014

	Methodological quality criteria	Responses			
		Yes	No	Can't tell	Comments
Screening questions	S1. Are there clear research questions?	x			
	S2. Do the collected data allow to address the research questions?	x			Though limited for some aspects
Quantitative descriptive	4.1. Is the sampling strategy relevant to address the research question?	x			Convenience volunteer sample
	4.2. Is the sample representative of the target population?			x	Participation rate unknown
	4.3. Are the measurements appropriate?	x			Though some relevant omissions
	4.4. Is the risk of nonresponse bias low?			x	Unclear acceptance rate/non-participant rate or attributes
	4.5. Is the statistical analysis appropriate to answer the research question?	x			

Morosini et al., 2014

	Methodological quality criteria	Responses			
		Yes	No	Can't tell	Comments
Screening questions	S1. Are there clear research questions?	x			
	S2. Do the collected data allow to address the research questions?	x			
Quantitative descriptive	4.1. Is the sampling strategy relevant to address the research question?	x			
	4.2. Is the sample representative of the target population?			x	But not aim of study
	4.3. Are the measurements appropriate?	x			
	4.4. Is the risk of nonresponse bias low?			x	But not aim of study
	4.5. Is the statistical analysis appropriate to answer the research question?	x			

Muniz et al., 2022

	Methodological quality criteria	Responses			
		Yes	No	Can't tell	Comments
Screening questions	S1. Are there clear research questions?	x			
	S2. Do the collected data allow to address the research questions?	x			Though limited for some associated factors
Quantitative descriptive	4.1. Is the sampling strategy relevant to address the research question?	x			Convenience volunteer sample
	4.2. Is the sample representative of the target population?	x			Highly likely as 92% participation rate
	4.3. Are the measurements appropriate?	x			Included variable measurements appropriate, but some likely associated factors absent
	4.4. Is the risk of nonresponse bias low?	x			92% participation rate
	4.5. Is the statistical analysis appropriate to answer the research question?	x			Within limitations of data collected

Muñoz et al., 2024

	Methodological quality criteria	Responses			
		Yes	No	Can't tell	Comments
Screening questions	S1. Are there clear research questions?	x			
	S2. Do the collected data allow to address the research questions?	x			
Quantitative descriptive	4.1. Is the sampling strategy relevant to address the research question?	x			Convenience volunteer sample
	4.2. Is the sample representative of the target population?	x			Highly likely as 92% participation rate
	4.3. Are the measurements appropriate?	x			
	4.4. Is the risk of nonresponse bias low?	x			92% participation rate
	4.5. Is the statistical analysis appropriate to answer the research question?	x			Limited to dichotomous caries/DMFT groups

Oliveria et al., 2015

	Methodological quality criteria	Responses			
		Yes	No	Can't tell	Comments
Screening questions	S1. Are there clear research questions?	x			
	S2. Do the collected data allow to address the research questions?	x			
Quantitative descriptive	4.1. Is the sampling strategy relevant to address the research question?	x			Convenience volunteer sample
	4.2. Is the sample representative of the target population?			x	Unclear full cohort details and representation
	4.3. Are the measurements appropriate?	x			
	4.4. Is the risk of nonresponse bias low?			x	Full population details unclear
	4.5. Is the statistical analysis appropriate to answer the research question?		x		Dichotomisation validity often questionable

Oner Ozdas et al., 2018

	Methodological quality criteria	Responses			
		Yes	No	Can’t tell	Comments
Screening questions	S1. Are there clear research questions?	x			
	S2. Do the collected data allow to address the research questions?	x			
Quantitative descriptive	4.1. Is the sampling strategy relevant to address the research question?			x	Power analysis performed but participants not uniformly distributed between groups
	4.2. Is the sample representative of the target population?			x	Sampling strategy not described
	4.3. Are the measurements appropriate?	x			
	4.4. Is the risk of nonresponse bias low?			x	Sampling strategy not described
	4.5. Is the statistical analysis appropriate to answer the research question?	x			However rudimentary and not including all data recorded

Pallavi et al., 2018

	Methodological quality criteria	Responses			
		Yes	No	Can’t tell	Comments
Screening questions	S1. Are there clear research questions?	x			
	S2. Do the collected data allow to address the research questions?	x			Collected data additional to research question
Quantitative descriptive	4.1. Is the sampling strategy relevant to address the research question?	x			Sample size calculation performed; convenience volunteer sample
	4.2. Is the sample representative of the target population?			x	Details not provided but did state not all sites were represented
	4.3. Are the measurements appropriate?	x			Generally; some subjective/ill-defined criteria
	4.4. Is the risk of nonresponse bias low?			x	Details of non-participants not provided beyond “ill and uncooperative excluded”
	4.5. Is the statistical analysis appropriate to answer the research question?	x			Descriptive to answer research question but included additional analysis beyond this scope

Russel et al., 2006

	Methodological quality criteria	Responses			
		Yes	No	Can't tell	Comments
Screening questions	S1. Are there clear research questions?	x			
	S2. Do the collected data allow to address the research questions?	x			
Qualitative	1.1. Is the qualitative approach appropriate to answer the research question?	x			
	1.2. Are the qualitative data collection methods adequate to address the research question?	x			Focus groups
	1.3. Are the findings adequately derived from the data?		x		Appears more topic-related narrative than thematic analysis
	1.4. Is the interpretation of results sufficiently substantiated by data?	x			
	1.5. Is there coherence between qualitative data sources, collection, analysis and interpretation?		x		Specific sampling and/or data saturation details unclear; appears more topic-related narrative than thematic analysis

Silva et al., 2021

	Methodological quality criteria	Responses			
		Yes	No	Can't tell	Comments
Screening questions	S1. Are there clear research questions?	x			
	S2. Do the collected data allow to address the research questions?	x			Though limited for some associated factors
Quantitative descriptive	4.1. Is the sampling strategy relevant to address the research question?	x			Convenience volunteer sample
	4.2. Is the sample representative of the target population?	x			Highly likely as 92% participation rate
	4.3. Are the measurements appropriate?	x			Included variable measurements appropriate, but some likely associated factors absent
	4.4. Is the risk of nonresponse bias low?	x			92% participation rate
	4.5. Is the statistical analysis appropriate to answer the research question?	x			Within limitations of data collected

Van Harten et al., 2015

	Methodological quality criteria	Responses			
		Yes	No	Can't tell	Comments
Screening questions	S1. Are there clear research questions?	x			
	S2. Do the collected data allow to address the research questions?	x			Collected data additional to research question
Quantitative descriptive	4.1. Is the sampling strategy relevant to address the research question?	x			Convenience volunteer sample
	4.2. Is the sample representative of the target population?			x	Participation rate of total population not provided (100% of eligible)
	4.3. Are the measurements appropriate?	x			
	4.4. Is the risk of nonresponse bias low?			x	Details of non-participants not provided beyond declined oral care services, unable to understand and those posing security risk
	4.5. Is the statistical analysis appropriate to answer the research question?		x		Descriptive caries data answers research question but only highest debris and calculus score data; additional analysis beyond this scope

Vinnerljung et al., 2018

	Methodological quality criteria	Responses			
		Yes	No	Can't tell	Comments
Screening questions	S1. Are there clear research questions?		x		Subjective level of dental health problem
	S2. Do the collected data allow to address the research questions?			x	Critical methodological information lacking
Quantitative descriptive	4.1. Is the sampling strategy relevant to address the research question?	x			Convenience volunteer sample with deliberate oversampling of females
	4.2. Is the sample representative of the target population?	x			Highly likely given initial 95% participant rate and only 5% attrition (oversampling of females)
	4.3. Are the measurements appropriate?		x		"Rudimentary" dental examination by minimally trained medical practitioner with no criteria defined
	4.4. Is the risk of nonresponse bias low?			x	Details of non-participants or those lost to attrition not provided
	4.5. Is the statistical analysis appropriate to answer the research question?	x			Within limitations of both research question and data collected (basic descriptive prevalence on dichotomous basis)

Zorlu et al., 2023

	Methodological quality criteria	Responses			
		Yes	No	Can't tell	Comments
Screening questions	S1. Are there clear research questions?	x			Broad
	S2. Do the collected data allow to address the research questions?		x		Cannot match breadth of research question
Quantitative descriptive	4.1. Is the sampling strategy relevant to address the research question?	x			Convenience volunteer sample
	4.2. Is the sample representative of the target population?			x	No sampling strategy or participation rate described
	4.3. Are the measurements appropriate?	x			Within limitations of data collected
	4.4. Is the risk of nonresponse bias low?			x	Acceptance rate/non-participant rate or attributes unknown
	4.5. Is the statistical analysis appropriate to answer the research question?		x		Rudimentary oral health status data presented with detail insufficient to answer treatment needs component of research question even for limited scope of measurements used
